# Superior vena cava syndrome with extensive collateral vessels

**DOI:** 10.1111/1759-7714.13225

**Published:** 2019-10-21

**Authors:** In‐Jae Oh, Cheol‐Kyu Park, Young‐Chul Kim

**Affiliations:** ^1^ Department of Internal Medicine Lung Cancer Clinic CNU, Chonnam National University Hwasun Hospital Hwasun Korea

**Keywords:** Chemotherapy, small cell lung carcinoma, superior vena cava syndrome

**Figure 1:**
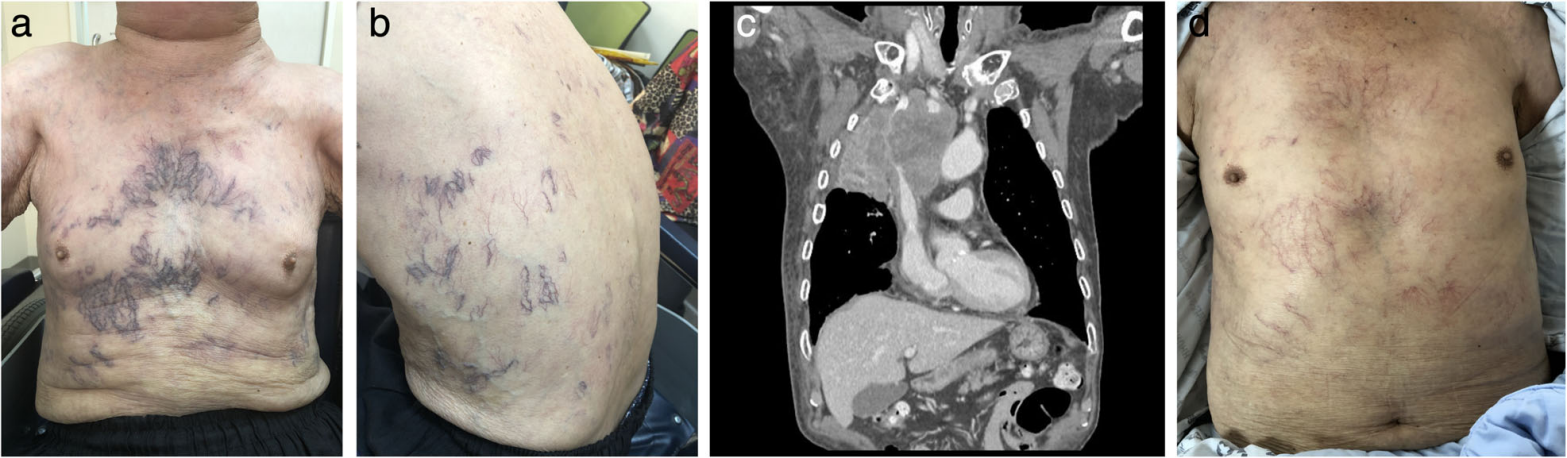
Anterior and Lateral Chest (a, b) and Computed Tomogram (c) before chemotherapy, and Chest (d) after chemotherapy.

An 84‐year‐old man visited a lung cancer clinic with a history of facial swelling of one week's duration. The patient was a 45 pack per year former smoker who had previously quit smoking 19 years ago. On examination, extensively dilated superficial vessels were noted on his torso (Fig 1a,b). Chest computed tomogram subsequently revealed almost complete occlusion of the superior vena cava by a huge mass in the right upper lung, together with enlarged mediastinal lymph nodes (Fig 1c). Chemotherapy with etoposide and cisplatin was administered following cervical lymph node biopsy and the diagnosis of small‐cell carcinoma. Nine days after the first dose of chemotherapy, the patient visited the emergency department and was diagnosed with febrile neutropenia. Figure 1d was taken on the 11th day after the first dose of chemotherapy and showed markedly decreased collateral vessels on his chest. This case demonstrates that complete relief of symptoms of vena caval obstruction can be achieved with chemotherapy in approximately 80% of patients with small‐cell lung cancer.

